# The Association of Physiotherapy Continuity of Care with Duration of Time Loss Among Compensated Australian Workers with Low Back Pain

**DOI:** 10.1007/s10926-024-10209-8

**Published:** 2024-05-25

**Authors:** Shannon E. Gray, Benedict Tudtud, Luke R. Sheehan, Michael Di Donato

**Affiliations:** Healthy Working Lives Research Group, School of Public Health and Preventive Medicine, Monash University, 553 St Kilda Rd, Melbourne, VIC 3004 Australia

**Keywords:** Low back pain, Physiotherapy, Continuity of care, Workers’ compensation

## Abstract

**Purpose:**

The aims of this study are to determine how continuous the care provided by physiotherapists to compensated workers with low back pain is, what factors are associated with physiotherapy continuity of care (CoC; treatment by the same provider), and what the association between physiotherapy CoC and duration of working time loss is.

**Methods:**

Workers’ compensation claims and payments data from Victoria and South Australia were analysed. Continuity of care was measured with the usual provider continuity metric. Binary logistic regression examined factors associated with CoC. Cox regression models examined the association between working time loss and CoC.

**Results:**

Thirty-six percent of workers experienced complete CoC, 25.8% high CoC, 26.1% moderate CoC, and 11.7% low CoC. Odds of complete CoC decreased with increased service volume. With decreasing CoC, there was significantly longer duration of compensated time loss.

**Conclusion:**

Higher CoC with a physiotherapist is associated with shorter compensated working time loss duration for Australian workers with low back pain.

**Supplementary Information:**

The online version contains supplementary material available at 10.1007/s10926-024-10209-8.

## Introduction

Continuity of care (CoC) is the provision of uninterrupted care by the same provider over time and is associated with improved patient outcomes [1]. Patients who experience more continuous care with the same provider report more positive patient experiences, greater patient satisfaction, higher treatment adherence, and improved health outcomes [1, 2]. Continuity of provider allows the patient–provider relationship to develop and promotes trust. It also means that “hand-overs” between healthcare providers can be avoided, limiting any information loss and ensuring treatment remains consistent.

Low back pain (LBP) is a common global health problem and is the leading cause of years lived with disability. Approximately 7.5% of the global population, or around 619 million people, suffered from LBP in 2020 [3]. LBP may result in significant economic and personal impacts, interfering with quality of life and performance at work [4]. People of working age are commonly impacted by LBP [5], with workers in many countries eligible for income support from workers’ compensation if they can prove a demonstrable link between LBP and activities of employment [6]. Workers’ compensation systems also fund healthcare. In cases of LBP, this often includes interactions with primary care providers such as general practitioners and physiotherapists.

Primary healthcare providers play critical roles in workers’ compensation systems. This includes certifying work capacity, coordinating other healthcare providers, and working with claims managers in addition to treating injured workers. Physiotherapists are common treatment providers for those with LBP. They can provide most of the recommended management options for LBP such as advice to remain active, avoid prolonged bed rest, and continue usual activities where possible for acute LBP. In addition, they can be educated about beneficial self-management options such as superficial heat [7–9]. For those at risk of chronic LBP (i.e. persisting beyond three months) or poor prognosis, exercise therapy with or without spinal manipulation may be recommended [7, 8].

A more continuous patient-provider relationship may lead the healthcare provider to take a more active role in coordinating healthcare, including development of a return to work plan or communication with case managers or employers [10]. Furthermore, an established relationship between a worker and their provider may negate the need for a worker to repeatedly explain to the healthcare provider their work capacity.

While an important topic, there is limited literature investigating outcomes associated with CoC in people with LBP. Van der Weide et al. (1999) found those workers with LBP who experienced higher CoC were associated with greater patient satisfaction, higher return to work rates at three months, and shorter overall time to return to work [11]. An Australian study found that workers with high CoC with a primary care physician were away from work for a shorter time, with this effect most prominent after being off work for one to two months [10]. Magel et al. (2018) undertook the only known study of physiotherapy CoC for those with LBP, and found that workers who experienced more continuous care with the same physical therapist had a lower likelihood of surgical intervention and reduced LBP-related healthcare costs [12].

It is hypothesised that physiotherapy-related CoC is also related to duration of working time loss and recovery; however, this relationship has not yet been explored. Thus, this study applies a CoC metric to workers’ compensation administrative claim data with payment-level data for all provided healthcare, allowing understanding of the relationship between CoC and recovery from LBP. In order to do so, this study has three research questions:

1. How continuous is the care provided by physiotherapists to compensated workers with LBP?
2. What are the demographic, occupational, and social factors associated with physiotherapy CoC among compensated workers with LBP?
3. What is the association between physiotherapy CoC for compensated workers with LBP and duration of working time loss?

## Methods

### Setting

Each of Australia’s 6 states and 2 territories have their own workers’ compensation system. There are also three national schemes for national employers and Commonwealth government employees, the military, and seafarers. All schemes provide wage replacement payments and cover ‘reasonable and necessary’ medical expenses and services for workers with accepted claims for injuries or illnesses suffered in the course of their employment. This often includes treatment provided by physiotherapists, who can be chosen at the worker’s discretion, provided the physiotherapist is registered with the workers’ compensation scheme.

### Data Source

The Monash University Multi-Jurisdictional Workers’ Compensation Database (MJD), which has been described previously, provided data for this study [13]. The MJD contains de-identified administrative workers’ compensation claims and associated service payments data for musculoskeletal conditions from five of Australia’s workers’ compensation jurisdictions, with injury dates ranging from 1 July 2010 to 30 June 2015.

### Inclusion Criteria

Accepted workers’ compensation claims for LBP, with a claim acceptance date between 1 July 2011 and 30 June 2015, were included. LBP claims were defined using Type of Occurrence Classification System version 3.1 nature and location of injury codes (see Supplementary Table 1) [14]. Claims from Victoria and South Australia were included as both jurisdictions contained a unique and de-identified code for each treating physiotherapist (not clinic), enabling quantification of services provided by the same physiotherapist. These two jurisdictions comprised 32% of the Australian labour force at the mid-point of this study, 2013 [15]. Claims with fewer than four physiotherapy encounters services were excluded, consistent with other CoC studies [10, 16–18]. Cases with missing covariate information were removed (*n* = 321).

### Physiotherapy Encounters

Physiotherapy encounters were defined as interactions between an injured worker and a physiotherapist. Payments for report writing, review of reports or programs, supplies or equipment, or for patient non-attendance were excluded. Duplicate services were removed (e.g. no more than one encounter per worker per day).

### Continuity of Care

Continuity of care with a physiotherapist was measured using the Usual Provider Continuity (UPC) index, as it is the most direct measure of the relationship between a worker and their ‘usual’ physiotherapist [19]. Calculated as the proportion of physiotherapist encounters that were with the most frequently seen service provider, the UPC has a range from $\frac{1}{n}$ (all services with different physiotherapists) to 1 (all services with the same physiotherapist). The following categories of CoC were used, in line with previous analyses [10, 18]: Complete CoC (UPC = 1), High CoC (UPC score of 0.75–0.99), Moderate CoC (UPC score of 0.5–0.74), and Low CoC (UPC < 0.5).

### Working Time Loss

Working time loss was defined as the cumulative number of calendar weeks of income support payments paid. Time loss duration was right censored at 104 weeks to remain consistent with other time loss analyses of worker’s compensation [20, 21].

### Covariates

Physiotherapist encounter count was categorised into quartiles for analysis with four service groups defined: Low service group (4–8 physiotherapy encounters), Moderate service group [9–18 physiotherapy encounters], High service group (19–36 physiotherapy encounters), and Very High service group (37+ physiotherapy encounters). Sex was recorded as either male or female. Age was categorised into 10-year groups, with those aged over 56 years combined into one category to account for small numbers. Jurisdiction refers to the state in which the worker made their workers’ compensation claim. Occupation was coded at major group level as defined by the Australian and New Zealand Standard Classification of Occupations [22]. The Australian Statistical Geography Standard was matched to residential postcode to define remoteness, with outer regional, remote, and very remote combined into a single group due to low frequencies [23].

### Analysis

A description of the cohort was tabulated overall and by UPC category, showing frequency and row percentage. Total within each UPC category was also generated, along with row percentage.

UPC categories were re-grouped into three new binary outcome variables to determine factors associated with UPC score: Low CoC versus Moderate, High, or Complete CoC; Moderate or Low CoC versus High or Complete CoC; and High, Moderate, or Low CoC versus Complete CoC [18]. These were estimated using three separate binary logistic regression models, including all covariates listed above, as these were hypothesised to have a relationship with UPC score. Ordinal logistic regression was not used as odds were not proportional. The reference was the covariate category with the highest frequency. Results were reported as odds ratios with corresponding 95% confidence intervals.

**Fig.1:**
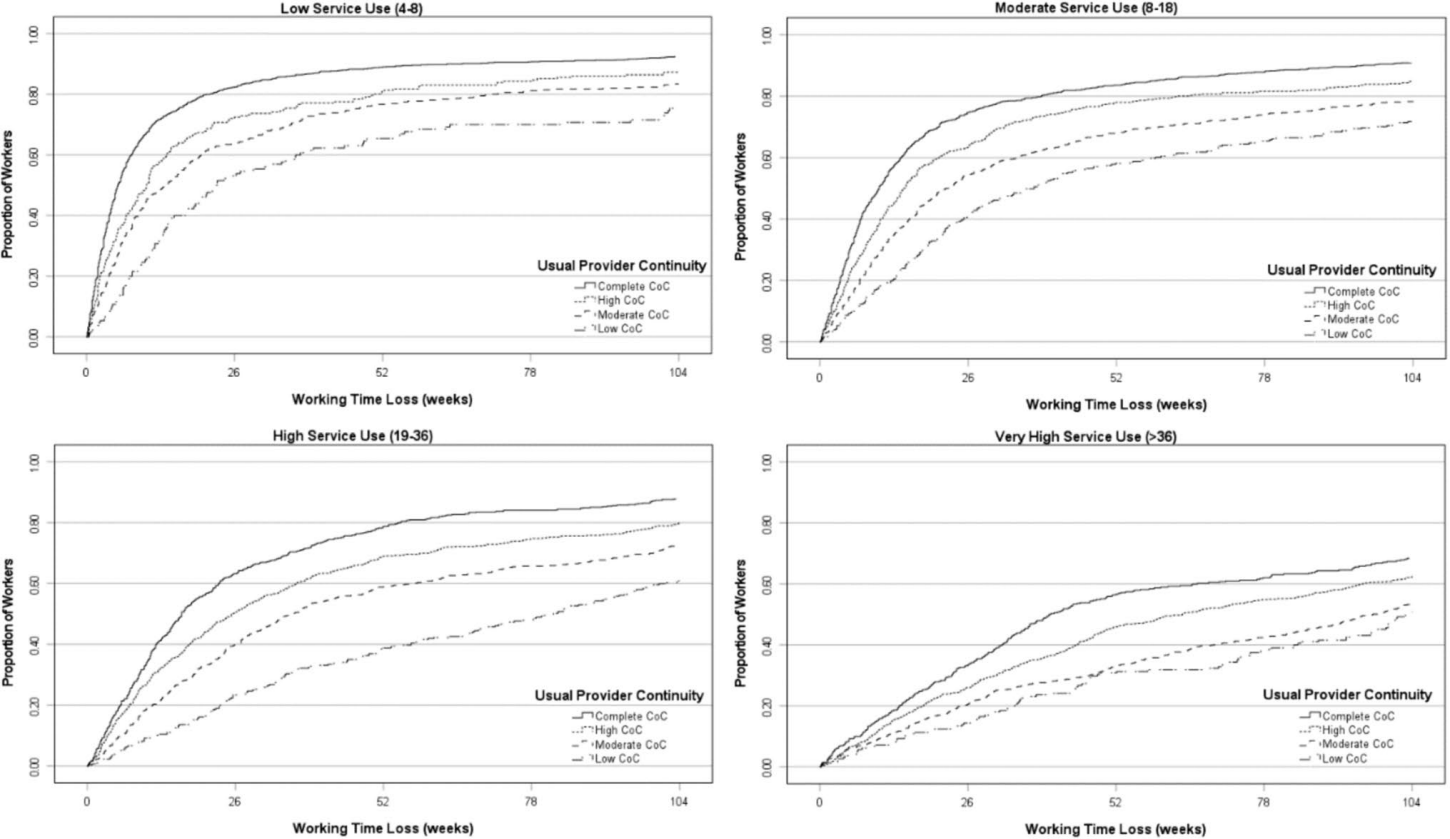
Duration of working time loss by usual provider category (UPC) and physiotherapy service use quartile

Kaplan–Meier failure plots, which show the cumulative proportion of workers by their time loss duration, were used to assess the relationship between CoC and working time loss stratified by service use quartile. The Kaplan–Meier plots (Fig. 1) indicate that the relationship between UPC category and working time loss was proportional over time, and thus, Cox regression was considered appropriate.

Median weeks’ time loss was tabulated with corresponding interquartile ranges. The outcome for Cox regression was the right-censored working time loss variable, using UPC category as the predictor and all covariates from above also included. Hazard ratios were reported with corresponding 95% confidence intervals, with a value less than one representing longer duration of time loss than the reference. Statistical significance was considered where *p* < 0.05. Cox regression models stratifying for service use groups were also generated, and presented alongside overall results. This was an attempt to disentangle the effect of service volume on both CoC and time loss, and thus compare the model overall with those when stratified.

All statistical analysis was performed using IBM SPSS version 26 (Armonk, NY: IBM Corp). The Monash University Human Research Ethics Committee (Project ID: 17267, November 2018) provided ethical approval.

### Sensitivity Analysis

A sensitivity analysis comparing the use of the Bice-Boxerman Continuity of Care Index (COCI) with the UPC as a measure of CoC was conducted. The UPC measures only the continuity with the most common provider, whereas the COCI metric measures the dispersal of services among different providers [24]. The COCI was categorised in the same way as the UPC as conducted previously [18], and differences in results were noted in the results section with tables included in Supplementary Materials.

## Results

There were 7748 claims with at least four physiotherapy services (Table 1). Seventy percent were from Victoria (*n* = 5601), most were from males (*n* = 4903, 63.3%), and a quarter were from Labourers (*n* = 1909, 24.6%). More than one-third experienced complete CoC (*n* = 2849, 36.8%), with approximately one quarter each for moderate and high CoC, and 11.1% experienced low CoC (n = 862). A higher proportion of all claims from Victoria received high or complete CoC compared to South Australia. Supplementary Table 2 shows that more claims were categorised as having lower CoC when using the COCI metric.

**Table 1:** Sample characteristics

	Low CoC, UPC < 0.5		Moderate CoC, UPC 0.5–0.74		High CoC, UPC 0.75–0.99		Complete CoC, UPC 1.0		Total
	*N*	Row %	*N*	Row %	*N*	Row %	*N*	Row %	*N*
UPC category	862	11.1	2012	26.0	2025	26.1	2849	36.8	7748
No. physiotherapy services (quartiles)									
Low (4–8)	120	7.2	337	20.1	226	13.5	995	59.3	1678
Moderate (9–18)	265	12.6	520	24.8	426	20.3	890	42.4	2101
High (19–36)	297	14.8	571	28.5	565	28.2	574	28.6	2007
Very High (> 36)	180	9.2	584	29.8	808	41.2	390	19.9	1962
Age group									
15–25 years	101	12.9	192	24.5	186	23.7	305	38.9	784
26–35 years	224	12.4	509	28.2	480	26.6	589	32.7	1802
36–45 years	242	11.5	559	26.7	563	26.9	732	34.9	2096
46–55 years	209	10.4	504	25.0	543	26.9	761	37.7	2017
56+ years	86	8.2	248	23.6	253	24.1	462	44.0	1049
Sex									
Female	316	11.1	778	27.3	772	27.1	979	34.4	2845
Male	546	11.1	1234	25.2	1253	25.6	1870	38.1	4903
Jurisdiction									
South Australia	340	15.8	607	28.3	425	19.8	775	36.1	2147
Victoria	522	9.3	1405	25.1	1600	28.6	2074	37.0	5601
Occupation									
Clerical and administrative workers	21	11.4	58	31.4	47	25.4	59	31.9	185
Community and personal service workers	155	11.2	367	26.6	368	26.6	492	35.6	1382
Machinery operators and drivers	177	11.6	394	25.8	381	25.0	574	37.6	1526
Managers	53	13.1	110	27.2	102	25.2	140	34.6	405
Professionals	71	11.4	158	25.4	183	29.5	209	33.7	621
Sales workers	47	14.1	109	32.7	78	23.4	99	29.7	333
Technicians and trades workers	145	10.5	355	25.6	359	25.9	528	38.1	1387
Labourers	193	10.1	461	24.1	507	26.6	748	39.2	1909
Remoteness									
Major cities of Australia	708	12.2	1554	26.7	1543	26.5	2014	34.6	5819
Inner regional Australia	117	8.0	362	24.7	381	26.0	603	41.2	1463
Outer regional/remote/very remote Australia	37	7.9	96	20.6	101	21.7	232	49.8	466

*CoC* continuity of care, *UPC* usual provider continuity

Table 2 shows older workers (46+ years) and those living outside of major cities had lower odds of having low, low/moderate, or low/moderate/high CoC. Worker sex was not significant. Managers had higher odds of low CoC compared to Labourers, and Managers and Sales Workers also had higher odds of low/moderate CoC. Workers from South Australia had higher odds of low, low/moderate, or low/moderate/high CoC compared to Victorian workers.

**Table 2:** Multivariate binary logistic regression models with usual provider continuity category as the outcomes

	Low CoC (versus moderate, high or complete CoC)		Moderate or low CoC (versus high or complete CoC)		High, moderate, or Low CoC (versus complete CoC)	
	OR (95% CI)	*p*-value	OR (95% CI)	*p*-value	OR (95% CI)	*p*-value
No. physiotherapy services (quartiles)						
Low (4–8)	0.51 (0.41, 0.64)	< 0.001	0.61 (0.53, 0.70)	< 0.001	0.50 (0.44, 0.57)	< 0.001
Moderate (9–18)	Ref		Ref		Ref	
High (19–36)	1.26 (1.05, 1.51)	0.012	1.32 (1.16, 1.50)	< 0.001	1.88 (1.65, 2.14)	< 0.001
Very High (37 +)	0.74 (0.61, 0.91)	0.005	1.12 (0.98, 1.28)	0.085	3.08 (2.67, 3.55)	< 0.001
Age group						
15–25 years	1.12 (0.87, 1.44)	0.388	0.97 (0.81, 1.15)	0.716	0.91 (0.76, 1.09)	0.309
26–35 years	1.06 (0.87, 1.29)	0.564	1.10 (0.96, 1.25)	0.163	1.11 (0.97, 1.28)	0.130
36–45 years	Ref		Ref		Ref	
46–55 years	0.89 (0.73, 1.08)	0.239	0.86 (0.76, 0.98)	0.025	0.82 (0.71, 0.93)	0.003
56+ years	0.70 (0.54, 0.91)	0.008	0.77 (0.65, 0.90)	0.001	0.66 (0.56, 0.77)	< 0.001
Sex						
Male	Ref		Ref		Ref	
Female	0.96 (0.80, 1.16)	0.694	1.07 (0.95, 1.21)	0.252	1.09 (0.97, 1.24)	0.156
Jurisdiction						
South Australia	1.96 (1.68, 2.29)	< 0.001	1.68 (1.51, 1.87)	< 0.001	1.39 (1.24, 1.56)	< 0.001
Victoria	Ref		Ref		Ref	
Occupation						
Clerical and administrative workers	1.10 (0.67, 1.79)	0.703	1.37 (1.00, 1.88)	0.048	1.35 (0.96, 1.90)	0.087
Community and personal service workers	1.14 (0.89, 1.45)	0.296	1.14 (0.97, 1.33)	0.106	1.15 (0.98, 1.35)	0.098
Machinery operators and drivers	1.11 (0.89, 1.39)	0.356	1.13 (0.98, 1.31)	0.102	1.07 (0.92, 1.24)	0.400
Managers	1.42 (1.02, 1.98)	0.036	1.31 (1.04, 1.63)	0.020	1.17 (0.92, 1.48)	0.205
Professionals	1.28 (0.94, 1.74)	0.117	1.13 (0.93, 1.38)	0.222	1.18 (0.96, 1.46)	0.119
Sales workers	1.35 (0.94, 1.92)	0.100	1.53 (1.20, 1.95)	0.001	1.32 (1.01, 1.72)	0.044
Technicians and trades workers	0.96 (0.76, 1.21)	0.739	1.04 (0.90, 1.21)	0.608	1.02 (0.87, 1.18)	0.841
Labourers	Ref		Ref		Ref	
Remoteness						
Major cities of Australia	Ref		Ref		Ref	
Inner regional Australia	0.68 (0.55, 0.83)	< 0.001	0.83 (0.74, 0.95)	0.004	0.88 (0.78, 1.00)	0.052
Outer regional/remote/very remote Australia	0.55 (0.39, 0.78)	0.001	0.60 (0.48, 0.74)	< 0.001	0.61 (0.50, 0.75)	< 0.001

*CoC* continuity of care, *OR* odds ratio

Those with low or very high service use had significantly lower odds of low CoC than those with moderate service use. Low service use also had lower odds for low/moderate and low/moderate/high CoC. Those with high service use had 32% higher odds of low/moderate CoC than moderate service use. Only when considering low/moderate/high CoC as the outcome was there a consistent relationship between increasing service use and odds ratios.

There were no changes to the statistical significance of the model coefficients between UPC and COCI metrics for logistic regression output (Supplementary Table 3).

Median time loss was shortest for workers with complete CoC (11.1 weeks, IQR 4.0–35.4) and longest for workers with low CoC (62.2 weeks, IQR 20.1–120.9). Median time loss increased as CoC decreased, and Cox regression showed this was significant overall and when stratified by service use category (Table 3). Overall, compared to the reference group (complete CoC), there was significantly longer duration of time loss for workers with high CoC (HR: 0.70, 95% CI 0.66–0.75), moderate CoC (HR: 0.53, 95% CI 0.50–0.57), and low CoC (HR: 0.36, 95% CI 0.33–0.40). Full results (including all covariates) are presented in Supplementary Table 5. The only difference between UPC and COCI (Supplementary Table 4) was that for the low service use group, the hazard ratio for high CoC was lower than for moderate CoC (meaning time loss did not increase as CoC decreased).

**Table 3:** Median duration of working time loss and difference in time loss estimated using Cox regression, overall and by service use category

	Median week's time loss (IQR)	Overall		Low (4–8)		Moderate (9–18)		High (19–36)		Very High (> 36)	
		HR (95% CI)	*p*-value	HR (95% CI)	*p*-value	HR (95% CI)	*p*-value	HR (95% CI)	*p*-value	HR (95% CI)	*p*-value
UPC category											
Low CoC, UPC < 0.5	62.2 (20.1, 120.9)	0.36 (0.33, 0.40)	< 0.001	0.34 (0.27, 0.42)	< 0.001	0.39 (0.33, 0.45)	< 0.001	0.33 (0.27, 0.39)	< 0.001	0.51 (0.40, 0.65)	< 0.001
Moderate CoC, UPC 0.5–0.74	36.4 (11.6, 113.8)	0.53 (0.50, 0.57)	< 0.001	0.56 (0.49, 0.64)	< 0.001	0.53 (0.47, 0.59)	< 0.001	0.53 (0.46, 0.60)	< 0.001	0.56 (0.47, 0.66)	< 0.001
High CoC, UPC 0.75–0.99	30.4 (10.0, 104.0)	0.70 (0.66, 0.75)	< 0.001	0.64 (0.54, 0.74)	< 0.001	0.73 (0.64, 0.82)	< 0.001	0.69 (0.61, 0.79)	< 0.001	0.76 (0.65, 0.88)	< 0.001
Complete CoC, UPC 1	11.1 (4.0, 35.4)	Ref		Ref		Ref		Ref		Ref	

*A HR* < *1 indicates greater duration of time loss and *vice versa

*UPC,* usual provider continuity, *CoC* continuity of care, *HR* hazard ratio, *CI* confidence interval

## Discussion

Our study investigated the relationship between physiotherapy continuity of care and duration of working time loss in Australian workers with accepted workers’ compensation claims for low back pain. We found that more than a third (36.4%) experienced complete CoC, and that there was a trend for shorter durations of working time loss with increasing levels of CoC. Those with high physiotherapy service use (19–36 services) and younger workers had higher odds of low CoC, whereas older workers, those with lower physiotherapy use, and those from Victoria or outer regional or remote areas were more likely to have complete CoC. The results show that being managed by the same physiotherapist is associated with shorter durations of working time loss, and may help recovery and return to work in a workers’ compensation setting.

A possible explanation for the association between continuous physiotherapy care with the same provider and work disability duration is that lower CoC increases the risk of workers receiving conflicting treatment or advice when encountering multiple physiotherapists, even if they are within the same clinic. Furthermore, with each new physiotherapist the worker must explain their condition, their current or past treatment, and their recovery. This requirement to re-explain the details surrounding the LBP to different providers may result in the worker having to “prove” they are in pain, hampering recovery [25]. Finally, there is always a chance that the worker and physiotherapist will not build rapport, and thus the worker may be less inclined to follow their advice or receive non-evidence-based treatment and advice, potentially stalling recovery and return to work.

Magel et al. highlighted that lower CoC with physical therapists is associated with negative outcomes, such as higher incidence of surgery and increased healthcare costs [12]. They postulate that receiving care from fewer physical therapist providers means more coherent management that involves fewer management strategies, and that with lower CoC a patient’s condition may worsen to the point where surgery is the necessary intervention, also increasing costs. Surgeons may then have their preferred physiotherapists, further reducing CoC.

There was a similar proportion of workers with complete CoC when encountering physiotherapists and primary care physicians (when comparing studies using similar methodologies) [10]; however, in general, CoC was higher among primary care physicians than physiotherapists. This could be due to workers being more likely to have an existing relationship with a (“regular”) primary care physician as they are the first point of treatment for most conditions, whereas the need to see a physiotherapist would likely only arise in the event of injury. Regardless of treatment provider, however, the relationship between higher CoC and shorter duration of working time loss was consistent [10].

Another consideration is that we may be observing lower CoC from workers who are dissatisfied with the physiotherapist they are seeing, or are unhappy with the way their recovery is progressing, making them more likely to seek out different physiotherapists. Moreover, clinical practice guidelines recommend exercise with or without spinal manipulation [7, 8], and for those not receiving spinal manipulation or ‘hands-on’ treatment may feel as though their physiotherapist is not helping them, causing them to seek other options. Alternatively, workers may be encouraged to seek second opinions or referral to more experienced practitioners (including pain physiotherapists) where recovery is not progressing, reducing CoC.

In contrast to the CoC study of primary care physicians, workers from major cities had lower CoC. There are fewer allied health professionals per person in regional Australia, potentially meaning it was not possible to choose provider or there were limited choices [26]. Older workers were more likely to have complete CoC. It is possible they have already encountered a physiotherapist over the course of their lifetime for an unrelated condition and thus found one they trust, compared to younger workers. CoC was significantly higher in Victoria compared to South Australia.

Our study benefited from a large multi-jurisdiction sample of workers’ compensation claims. Combining claim and service-level data (that included a unique code for each physiotherapist) allowed analysis of outcomes that have not previously been measured in Australian workers’ compensation. The Usual Provider Continuity metric was considered a robust measure of CoC from previous work [10], and sensitivity analysis revealed no significant differences from the Bice-Boxerman COCI metric. However, these metrics do not consider any services paid for outside of the workers’ compensation system (e.g. by the worker themselves) but we do know they are for treatment of the same condition. We also could not account for any non-physiotherapy treatment that could support or hinder recovery. Further, it does not capture how the worker experiences CoC (e.g. relationships with different providers, transfer of information between providers). Covariates included those available in administrative data; however, there are likely more covariates that impact CoC, so residual confounding is possible. Finally, working time loss is cumulative, so for part-time workers or those who return to work partially the measure may not accurately reflect their time off work. For the purposes of Cox regression, we assumed that there was only one return to work event, which may not be valid for every worker as it fails to consider relapses.

## Conclusion

Experiencing more continuous care with the same physiotherapist is associated with reduced time off work for workers with accepted workers’ compensation claims for LBP. This relationship persisted after adjusting for other covariates known to be associated with duration of time loss, such as age, sex, jurisdiction, and occupation. Findings would be of value to workers’ compensation insurers; however, future research with both existing and additional data that seeks to integrate patterns of physiotherapy use and clinical pathways, including referrals, would be valuable.

## Supplementary Information

Below is the link to the electronic supplementary material.

Supplementary file1 (DOCX 22 kb)

## References

[1] Jackson C, Ball L. Continuity of care: vital, but how do we measure and promote it? Aust J Gen Pract. 2018;47(10):662–664. 31195766 10.31128/AJGP-05-18-4568

[2] Fuertes JN, Mislowack A, Bennett J, Paul L, Gilbert TC, Fontan G, et al. The physician-patient working alliance. Patient Educ Couns. 2007;66(1):29–36. 17188453 10.1016/j.pec.2006.09.013

[3] Ferreira ML, de Luca K, Haile LM, Steinmetz JD, Culbreth GT, Cross M, et al. Global, regional, and national burden of low back pain, 1990–2020, its attributable risk factors, and projections to 2050: a systematic analysis of the global burden of disease study 2021. Lancet Rheumatol. 2023;5(6):e316–e329. 37273833 10.1016/S2665-9913(23)00098-X PMC10234592

[4] Ehrlich GE. Low back pain. Bull World Health Organ. 2003;81(9):671–676. 14710509 PMC2572532

[5] Vos T, Lim SS, Abbafati C, Abbas KM, Abbasi M, Abbasifard M, et al. Global burden of 369 diseases and injuries in 204 countries and territories, 1990–2019: a systematic analysis for the global burden of disease study 2019. Lancet. 2020;396(10258):1204–1222. 33069326 10.1016/S0140-6736(20)30925-9 PMC7567026

[6] Oakman J, Clune S, Stuckey R. Work-related musculoskeletal disorders in Australia. Canberra: Safe Work Australia; 2019.

[7] Corp N, Mansell G, Stynes S, Wynne-Jones G, Morsø L, Hill JC, et al. Evidence-based treatment recommendations for neck and low back pain across Europe: a systematic review of guidelines. Eur J Pain. 2021;25(2):275–295. 33064878 10.1002/ejp.1679 PMC7839780

[8] Oliveira CB, Maher CG, Pinto RZ, Traeger AC, Lin C-WC, Chenot J-F, et al. Clinical practice guidelines for the management of non-specific low back pain in primary care: an updated overview. Eur Spine J. 2018. 10.1007/s00586-018-5673-2. 29971708 10.1007/s00586-018-5673-2

[9] Australian Physiotherapy Association (APA). What is Physio? 2023

[10] Sheehan LR, Di Donato M, Gray SE, Lane TJ, van Vreden C, Collie A. The association between continuity of care with a primary care physician and duration of work disability for low back pain: a retrospective cohort study. J Occup Environ Med/Am Coll Occup Environ Med. 2022;64(10):e606–e612. 10.1097/JOM.0000000000002643 35901194

[11] van der Weide WE, Verbeek JH, van Dijk FJ. Relation between indicators for quality of occupational rehabilitation of employees with low back pain. Occup Environ Med. 1999;56(7):488–493. 10472321 10.1136/oem.56.7.488 PMC1757763

[12] Magel J, Kim J, Thackeray A, Hawley C, Petersen S, Fritz JM. Associations between physical therapy continuity of care and health care utilization and costs in patients with low back pain: a retrospective cohort study. Phys Ther. 2018;98(12):990–999. 30260429 10.1093/ptj/pzy103

[13] Di Donato M, Sheehan L, Gray SE, Iles R, Van Vreden C, Collie A. Development and initial application of a harmonised multi-jurisdiction work injury compensation database. Digit Health. 2023. 10.1177/20552076231176695. 37312940 10.1177/20552076231176695 PMC10259130

[14] Australian Safety and Compensation Council. Type of Occurrence Classification System 3rd Edition, Revision 1. Canberra; 2008.

[15] Australian Bureau of Statistics. Labour Force, Australia (January 2013) Canberra, Australia: Australian Bureau of Statistics; 2013 updated February 7 2013. https://www.ausstats.abs.gov.au/ausstats/meisubs.nsf/0/5F3F7E6AA336A5E2CA257B0A000ECF16/$File/62020_jan%202013.pdf.

[16] Amjad H, Carmichael D, Austin AM, Chang CH, Bynum JP. Continuity of care and health care utilization in older adults with dementia in fee-for-service medicare. JAMA Intern Med. 2016;176(9):1371–1378. 27454945 10.1001/jamainternmed.2016.3553 PMC5061498

[17] Nyweide DJ, Anthony DL, Bynum JP, Strawderman RL, Weeks WB, Casalino LP, et al. Continuity of care and the risk of preventable hospitalization in older adults. JAMA Intern Med. 2013;173(20):1879–1885. 24043127 10.1001/jamainternmed.2013.10059 PMC3877937

[18] Tran B, Falster M, Jorm L. Claims-based measures of continuity of care have non-linear associations with health: data linkage study. Int J Popul Data Sci. 2018;3(1):463. 34095520 10.23889/ijpds.v3i1.463 PMC8142963

[19] Breslau N, Reeb KG. Continuity of care in a university-based practice. J Med Educ. 1975;50(10):965–969. 1159765 10.1097/00001888-197510000-00006

[20] Collie A, Lane TJ, Hassani-Mahmooei B, Thompson J, McLeod C. Does time off work after injury vary by jurisdiction? A comparative study of eight Australian workers’ compensation systems. BMJ Open. 2016;6(5):e010910. 27150186 10.1136/bmjopen-2015-010910 PMC4861102

[21] Di Donato M, Iles R, Buchbinder R, Xia T, Collie A. Prevalence, predictors and wage replacement duration associated with diagnostic imaging in Australian workers with accepted claims for low back pain: a retrospective cohort study. J Occup Rehabil. 2021;32(1):55–63. 33913056 10.1007/s10926-021-09981-8

[22] Australian Bureau of Statistics. 1220.0—ANZSCO—Australian and New Zealand Standard Classification of Occupations, 2013, Version 1.2. Canberra: Australian Bureau of Statistics; 2013.

[23] Australian Bureau of Statistics. 1270.0.55.006—Australian Statistical Geography Standard (ASGS): Correspondences, July 2011 Canberra: Australian Bureau of Statistics; 2012 http://www.abs.gov.au/AUSSTATS/abs@.nsf/DetailsPage/1270.0.55.006July%202011?OpenDocument.

[24] Bice TW, Boxerman SB. A quantitative measure of continuity of care. Med Care. 1977;15(4):347–349. 859364 10.1097/00005650-197704000-00010

[25] Hadler NM. If you have to prove you are ill, you can’t get well the object lesson of fibromyalgia. Spine. 1996;21(20):2397–2400. 10.1097/00007632-199610150-00021. 8915080 10.1097/00007632-199610150-00021

[26] Australian Institute of Health and Welfare. Health workforce Canberra, Australia: Australian Institute of Health and Welfare; 2022 updated 7 July 2022. https://www.aihw.gov.au/reports/workforce/health-workforce.

