# Supplementary material for: The Association of Physiotherapy Continuity of Care with Duration of Time Loss Among Compensated Australian Workers with Low Back Pain
Source: J Occup Rehabil. 2024 May 25;35(2):366–73. doi: 10.1007/s10926-024-10209-8 (PMC12089250; doi:10.1007/s10926-024-10209-8)
Supplement: Supplementary file 1 — Supplementary file1 (DOCX 22 kb) [file 10926_2024_10209_MOESM1_ESM.docx]

**Supplementary Table 1:** Low back pain selection criteria using Type of Occurrence Classification System 3^rd^ Edition

| **Nature of injury** | AND | **Location of injury** |
| --- | --- | --- |
| 228 – Trauma to muscles and tendons, not elsewhere classified OR 229 – Trauma to muscles and tendons, unspecified OR  239 – Soft tissue injuries due to trauma or unknown mechanisms with insufficient information to code OR  422 – Disc displacement, prolapse, degeneration or hernia OR  459 – Back pain, lumbago, or sciatica OR  533 – Muscle, tendon strain (non-traumatic) |  | 311 – Lower back |

**Supplementary Table 2:** Crosstabulation of usual provider continuity and Bice-Boxerman continuity of care index metrics

|  | | **COCI** | | | | **Total** |
| --- | --- | --- | --- | --- | --- | --- |
|  |  | **Low** | **Moderate** | **High** | **Complete** |  |
| **UPC** | **Low** | 862 | 0 | 0 | 0 | 862 |
|  | **Moderate** | 1304 | 708 | 0 | 0 | 2012 |
|  | **High** | 0 | 911 | 1114 | 0 | 2025 |
|  | **Complete** | 0 | 0 | 0 | 2849 | 2849 |
| **Total** | | 2313 | 2166 | 1619 | 1114 | 2849 |

*Note: COCI, Bice-Boxerman Continuity of Care Index; UPC, Usual Provider Continuity*

**Supplementary Table 3:** Multivariate binary logistic regression models with Bice-Boxerman categories as the outcomes

|  | **Low CoC (versus Moderate, High or Complete CoC)** | | **Moderate or Low CoC (versus High or Complete CoC)** | | **High, Moderate or Low CoC (versus Complete CoC)** | |
| --- | --- | --- | --- | --- | --- | --- |
|  | **OR (95% CI)** | **p-value** | **OR (95% CI)** | **p-value** | **OR (95% CI)** | **p-value** |
| **No. physiotherapy services (quartiles)** | | | | | | |
| Low (4-8) | 0.51 (0.41, 0.64) | <0.001 | 0.61 (0.53, 0.70) | <0.001 | 0.50 (0.44, 0.57) | <0.001 |
| Moderate (9-18) | Ref | | Ref | | Ref | |
| High (19-36) | 1.26 (1.05, 1.51) | 0.012 | 1.32 (1.16, 1.50) | <0.001 | 1.88 (1.65, 2.14) | <0.001 |
| Very High (>36) | 0.74 (0.61, 0.91) | 0.005 | 1.12 (0.98, 1.28) | 0.085 | 3.08 (2.67, 3.55) | <0.001 |
| **Age group** | | | | | | |
| 15-25 years | 1.12 (0.87, 1.44) | 0.388 | 0.97 (0.81, 1.15) | 0.716 | 0.91 (0.76, 1.09) | 0.309 |
| 26-35 years | 1.06 (0.87, 1.29) | 0.564 | 1.10 (0.96, 1.25) | 0.163 | 1.11 (0.97, 1.28) | 0.130 |
| 36-45 years | Ref | | Ref | | Ref | |
| 46-55 years | 0.89 (0.73, 1.08) | 0.239 | 0.86 (0.76, 0.98) | 0.025 | 0.82 (0.71, 0.93) | 0.003 |
| 56+ years | 0.70 (0.54, 0.91) | 0.008 | 0.77 (0.65, 0.90) | 0.001 | 0.66 (0.56, 0.77) | <0.001 |
| **Sex** | | | | | | |
| Male | Ref | | Ref | | Ref | |
| Female | 0.96 (0.80, 1.16) | 0.694 | 1.07 (0.95, 1.21) | 0.252 | 1.09 (0.97, 1.24) | 0.156 |
| **Jurisdiction** | | | | | | |
| South Australia | 1.96 (1.68, 2.29) | <0.001 | 1.68 (1.51, 1.87) | 0.000 | 1.39 (1.24, 1.56) | <0.001 |
| Victoria | Ref | | Ref | | Ref | |
| **Occupation** | | | | | | |
| Clerical and Administrative Workers | 1.10 (0.67, 1.79) | 0.703 | 1.37 (1.00, 1.88) | 0.048 | 1.35 (0.96, 1.90) | 0.087 |
| Community and Personal Service Workers | 1.14 (0.89, 1.45) | 0.296 | 1.14 (0.97, 1.33) | 0.106 | 1.15 (0.98, 1.35) | 0.098 |
| Machinery Operators and Drivers | 1.11 (0.89, 1.39) | 0.356 | 1.13 (0.98, 1.31) | 0.102 | 1.07 (0.92, 1.24) | 0.400 |
| Managers | 1.42 (1.02, 1.98) | 0.036 | 1.31 (1.04, 1.63) | 0.020 | 1.17 (0.92, 1.48) | 0.205 |
| Professionals | 1.28 (0.94, 1.74) | 0.117 | 1.13 (0.93, 1.38) | 0.222 | 1.18 (0.96, 1.46) | 0.119 |
| Sales Workers | 1.35 (0.94, 1.92) | 0.100 | 1.53 (1.20, 1.95) | 0.001 | 1.32 (1.01, 1.72) | 0.044 |
| Technicians and Trades Workers | 0.96 (0.76, 1.21) | 0.739 | 1.04 (0.90, 1.21) | 0.608 | 1.02 (0.87, 1.18) | 0.841 |
| Labourers | Ref | | Ref | | Ref | |
| **Remoteness** | | | | | | |
| Major Cities of Australia | Ref | | Ref | | Ref | |
| Inner Regional Australia | 0.68 (0.55, 0.83) | <0.001 | 0.83 (0.74, 0.95) | 0.004 | 0.88 (0.78, 1.00) | 0.052 |
| Outer Regional/Remote/Very Remote Australia | 0.55 (0.39, 0.78) | 0.001 | 0.60 (0.48, 0.74) | <0.001 | 0.61 (0.50, 0.75) | <0.001 |

*Note: CoC, continuity of care; OR, odds ratio; CI, confidence interval.*

**Supplementary Table 4:** Difference in time loss estimated using Cox regression, overall and by service use category (using Bice-Boxerman COCI metric)

|  | **Overall** | | **Low (4-8)** | | **Moderate (9-18)** | | **High (19-36)** | | **Very High (37+)** | |
| --- | --- | --- | --- | --- | --- | --- | --- | --- | --- | --- |
|  | **HR (95% CI)** | **p-value** | **HR (95% CI)** | **p-value** | **HR (95% CI)** | **p-value** | **HR (95% CI)** | **p-value** | **HR (95% CI)** | **p-value** |
| **COCI category** | | | | | | | | | | |
| Low CoC,  COCI < 0.5 | 0.45 (0.42, 0.47) | <0.001 | 0.46 (0.41, 0.53) | <0.001 | 0.46 (0.41, 0.52) | <0.001 | 0.41 (0.36, 0.47) | <0.001 | 0.52 (0.43, 0.62) | <0.001 |
| Moderate CoC,  COCI 0.5-0.74 | 0.63 (0.58, 0.67) | <0.001 | 0.68 (0.58, 0.79) | <0.001 | 0.62 (0.54, 0.70) | <0.001 | 0.59 (0.52, 0.68) | <0.001 | 0.65 (0.55, 0.77) | <0.001 |
| High CoC,  COCI 0.75-0.99 | 0.75 (0.69, 0.82) | <0.001 | 0.55 (0.41, 0.73) | <0.001 | 0.78 (0.66, 0.92) | 0.004 | 0.77 (0.67, 0.90) | 0.001 | 0.79 (0.67, 0.93) | 0.004 |
| Complete CoC,  COCI 1.0 | Ref | | Ref | | Ref | | Ref | | Ref | |

*Note: COCI, Bice-Boxerman Continuity of Care Index; CoC, continuity of care; HR, hazard ratio; CI, confidence interval. A HR < 1 indicates greater duration of time loss and vice-versa*

**Supplementary Table 5:** Median duration of working time loss and difference in time loss estimated using Cox regression

|  | **Median weeks' time loss (IQR)** | **HR (95% CI)** | **p-value** |
| --- | --- | --- | --- |
| **UPC category** | | | |
| Low CoC, UPC < 0.5 | 62.2 (20.1, 120.9) | 0.36 (0.33, 0.40) | <0.001 |
| Moderate CoC, UPC 0.5-0.74 | 36.4 (11.6, 113.8) | 0.53 (0.50, 0.57) | <0.001 |
| High CoC, UPC 0.75-0.99 | 30.4 (10.0, 104.0) | 0.70 (0.66, 0.75) | <0.001 |
| Complete CoC, UPC 1 | 11.1 (4.0, 35.4) | Ref | |
| **No. physiotherapy services (quartiles)** | | | |
| Low (4-8) | 8.0 (3.0, 25.0) | 1.31 (1.23, 1.41) | <0.001 |
| Moderate (9-18) | 15.3 (6.0, 49.6) | Ref | |
| High (19-36) | 29.0 (11.0, 97.6) | 0.73 (0.68, 0.78) | <0.001 |
| Very High (37+) | 72.7 (25.9, 125.1) | 0.44 (0.40, 0.47) | <0.001 |
| **Age group** | | | |
| 15-25 years | 15.9 (5.6, 52.3) | 1.33 (1.21, 1.46) | <0.001 |
| 26-35 years | 21.3 (7.0, 74.7) | 1.22 (1.13, 1.31) | <0.001 |
| 36-45 years | 26.4 (8.0, 102.9) | Ref | |
| 46-55 years | 26.6 (8.0, 103.9) | 0.98 (0.92, 1.06) | 0.662 |
| 56+ years | 24.6 (7.4, 104.3) | 0.96 (0.88, 1.05) | 0.351 |
| **Sex** | | | |
| Female | 21.4 (7.6, 66.7) | 1.16 (1.08, 1.23) | <0.001 |
| Male | 25.4 (7.0, 105.3) | Ref | |
| **Jurisdiction** | | | |
| South Australia | 14.0 (4.3, 61.7) | 1.52 (1.43, 1.61) | <0.001 |
| Victoria | 28.4 (9.1, 102.0) | Ref | |
| **Occupation** | | | |
| Clerical and Administrative Workers | 20.6 (6.1, 68.7) | 1.28 (1.08, 1.52) | 0.004 |
| Community and Personal Service Workers | 19.4 (6.7, 51.3) | 1.35 (1.24, 1.46) | <0.001 |
| Machinery Operators and Drivers | 25.8 (8.0, 110.6) | 1.05 (0.97, 1.13) | 0.274 |
| Managers | 30.3 (7.4, 112.9) | 1.06 (0.93, 1.20) | 0.382 |
| Professionals | 18.4 (7.4, 51.4) | 1.50 (1.35, 1.67) | <0.001 |
| Sales Workers | 20.4 (7.6, 78.7) | 1.26 (1.10, 1.43) | 0.001 |
| Technicians and Trades Workers | 24.7 (6.1, 104.4) | 1.06 (0.97, 1.15) | 0.187 |
| Labourers | 27.4 (8.3, 105.4) | Ref | |
| **Remoteness** | | | |
| Major Cities of Australia | 22.4 (7.0, 91.0) | Ref | |
| Inner Regional Australia | 27.6 (8.4, 99.3) | 0.79 (0.73, 0.84) | <0.001 |
| Outer Regional/Remote/Very Remote Australia | 29.0 (7.7, 115.1) | 0.62 (0.55, 0.69) | <0.001 |

*Note: UPC, usual provider continuity; CoC, continuity of care; HR, hazard ratio; CI, confidence interval. A HR < 1 indicates greater duration of time loss and vice-versa*
